# Protein Profiling of *Aedes aegypti* Treated with *Streptomyces* sp. KSF103 Ethyl Acetate Extract Reveals Potential Insecticidal Targets and Metabolic Pathways

**DOI:** 10.3390/ijms241512398

**Published:** 2023-08-03

**Authors:** Ker Shien Tan, Adzzie Shazleen Azman, Pouya Hassandarvish, Zheng Hua Amelia-Yap, Tiong Kai Tan, Van Lun Low

**Affiliations:** 1 Tropical Infectious Diseases Research and Education Centre (TIDREC), Universiti Malaya, Kuala Lumpur 50603, Malaysia; kershien99@gmail.com (K.S.T.); pouyahassandarvish@um.edu.my (P.H.); ame_lia1031@hotmail.com (Z.H.A.-Y.); 2 Institute for Advanced Studies (IAS), Universiti Malaya, Kuala Lumpur 50603, Malaysia; 3 School of Science, Monash University Malaysia, Bandar Sunway 47500, Malaysia; adzzieshazleen.azman@monash.edu; 4 Department of Parasitology, Faculty of Medicine, Universiti Malaya, Kuala Lumpur 50603, Malaysia; tantk@um.edu.my

**Keywords:** *Aedes aegypti*, proteomics, *Streptomyces*, liquid chromatography with tandem mass spectrometry, molecular docking

## Abstract

The insecticidal activity of *Streptomyces* sp. KSF103 ethyl acetate (EA) extract against mosquitoes is known; however, the underlying mechanism behind this activity remains elusive. In this study, liquid chromatography with tandem mass spectrometry (LC-MS/MS) was employed to investigate changes in the protein profile of *Aedes aegypti* larvae and adults treated with lethal concentrations of 50 (LC_50_) EA extract. By comparing the treated and untreated mosquitoes, this study aimed to identify proteins or pathways that exhibit alterations, potentially serving as targets for future insecticide development. Treatment with a lethal concentration of EA extract upregulated 15 proteins in larvae, while in adults, 16 proteins were upregulated, and two proteins were downregulated. These proteins were associated with metabolism, protein regulation/degradation, energy production, cellular organization and structure, enzyme activity, and catalysis, as well as calcium ion transport and homeostasis. Notably, ATP synthase, fructose-bisphosphate aldolase (FBA), and ATP citrate synthase were significantly expressed in both groups. Gene ontology analysis indicated a focus on energy metabolic processes. Molecular docking revealed a strong interaction between dodemorph, selagine (compounds from the EA extract), and FBA, suggesting FBA as a potential protein target for insecticide development. Further studies such as Western blot and transcriptomic analyses are warranted to validate the findings.

## 1. Introduction

The mosquito *Aedes aegypti*, which originated from the African subcontinent, is known to be one of the world’s deadliest animals [1]. It is the primary vector for four significant virus illnesses, specifically Chikungunya, dengue, yellow fever, and Zika. Due to its anthropophilic and breeding behavior, *Ae. aegypti* is recognized as a highly efficient vector of arboviruses in urban areas [2]. It also tends to bite multiple humans during the daytime to obtain complete blood meals and usually remains close to human populations (within 200 m) to ensure its blood source availability [3]. It preferentially breeds in clean or stagnant water located within buildings in suburban and urban areas, which increases the vector’s proximity to human hosts and may facilitate the spread of vector-borne viral diseases [4]. Besides arboviruses, *Ae. aegypti* is also responsible for the transmission of filarial parasites causing lymphatic filariasis [5]. Remarkably, *Aedes*-borne diseases have threatened human health, contributing to more than 50 million infections and approximately 300,000 deaths worldwide annually [6,7].

Insecticide application is one of the most effective approaches in vector control programs. Pyrethroid insecticides, for example, deltamethrin, cypermethrin, and permethrin, are commonly used against *Ae. aegypti* due to their low toxicity to mammals [8]. In addition, organophosphate insecticides, particularly temephos and malathion, are also frequently used to control *Ae. aegypti* larvae and adults [9]. Unfortunately, *Ae. aegypti* has reported resistance to four major classes of insecticides, including carbamates, organochlorines, organophosphate, and pyrethroids, due to the massive use of these chemical-based insecticide active ingredients [10,11]. The situation worsens with the elevation of environmental temperature caused by global warming, which further reduces the effectiveness of insecticides [12]. This, in turn, may have critical epidemiological effects on vector-borne diseases transmitted by *Ae. aegypti* [13], further underscoring the imperative of exploring alternative control methods.

Among the natural products derived from diverse microbes, the actinobacteria, namely streptomycetes, are a crucial source of valuable bioactive compounds. Recent scientific advancements have unveiled the antiviral, antibacterial, antifungal, antihypertensive, and antitumoral properties of streptomycetes, offering promising prospects for improving human health [14,15,16,17]. In the realm of pest control, several metabolites from the genus *Streptomyces*, for instance, prasinons and nanchangmycin have been used as potential active ingredients in biopesticide in response to the development of insecticide resistance in *Aedes* spp. [18,19]. Thus, screenings of natural agents from novel organisms, particularly *Streptomyces*, alongside the possibility of combining insecticides to combat resistant strains of mosquitoes, present an alternative approach to improve the effectiveness of vector control, thereby offering a remedy for vector-borne diseases.

*Streptomyces* sp. KSF103 isolated from bulk soils in the primary forest of Malaysia has been found to have insecticidal activities on various mosquitoes. This is a Gram-positive bacterium that exhibits an ingrowing wrinkled morphology and possesses the ability to produce spores while forming both aerial hyphae and substrate mycelia structures [20]. Based on the phylogenetic analysis using the 16S rRNA gene sequence, *Streptomyces* sp. KSF103 has the highest similarity with *Streptomyces rubrisoli* FXJ1.725 (99.18) and its nearly complete 16S rRNA sequence is accessible in GenBank under the accession number MT355788 [20]. While the killing effect is observed, the underlying mechanisms are not completely understood. Accordingly, this study aims to identify the protein profile of *Ae. aegypti* adults and larvae treated with *Streptomyces* sp. KSF103 ethyl acetate (EA) extract, and provide insights into the potential interaction between the bioactive compounds and mosquito proteins. The potential insecticide targets and metabolic pathways identified in this study might pave the way for important breakthroughs and advancements in the field of insecticide discovery.

## 2. Results

To examine the effect of EA extract on the protein profile of *Ae. aegypti*, the treated and untreated larval and adult samples were selected for proteomic analysis by LC-MS/MS analysis. This led to the identification of 189 proteins in pooled samples of the treatment group and 102 proteins in pooled samples of the control group during the larval stage. Both control and treatment groups shared 59 common proteins. In adults, a total of 670 proteins were determined in the treatment group while 515 proteins were in the control group. Of these, 385 were identical in both treatment and control samples.

Further filtration was applied to these similar proteins based on the cutoff score. A heatmap was performed to determine proteins that were differentially regulated between the protein samples from *Ae. aegypti* larvae and adults. In general, 15 proteins were significantly expressed in larval samples where all of them were upregulated (Supplementary Table S1). Heat shock cognate 70 exhibited the highest log_2_fold change (4.04-fold), followed by ubiquitin-60S ribosomal protein L40 (3.63-fold) and actin (3.46-fold). No significantly downregulated proteins were observed in larval samples. A total of 18 proteins were significantly altered in adult samples: 16 proteins were upregulated and 2 proteins were downregulated (Supplementary Table S1). The pyruvate kinase showed the most significant increase in log_2_fold change (3.62-fold), while AAEL009992-PA (3.05-fold) and 26S protease (S4) regulatory subunit (2.52-fold) exhibited slightly lower increases in expression. In contrast, the expression of alanine transaminase demonstrated the greatest downregulation in log_2_fold change (−1.79-fold), followed by 40S ribosomal protein 3a (−1.63-fold). The significantly expressed proteins were further examined by the UniProt database (Table 1).

**Figure 1:**
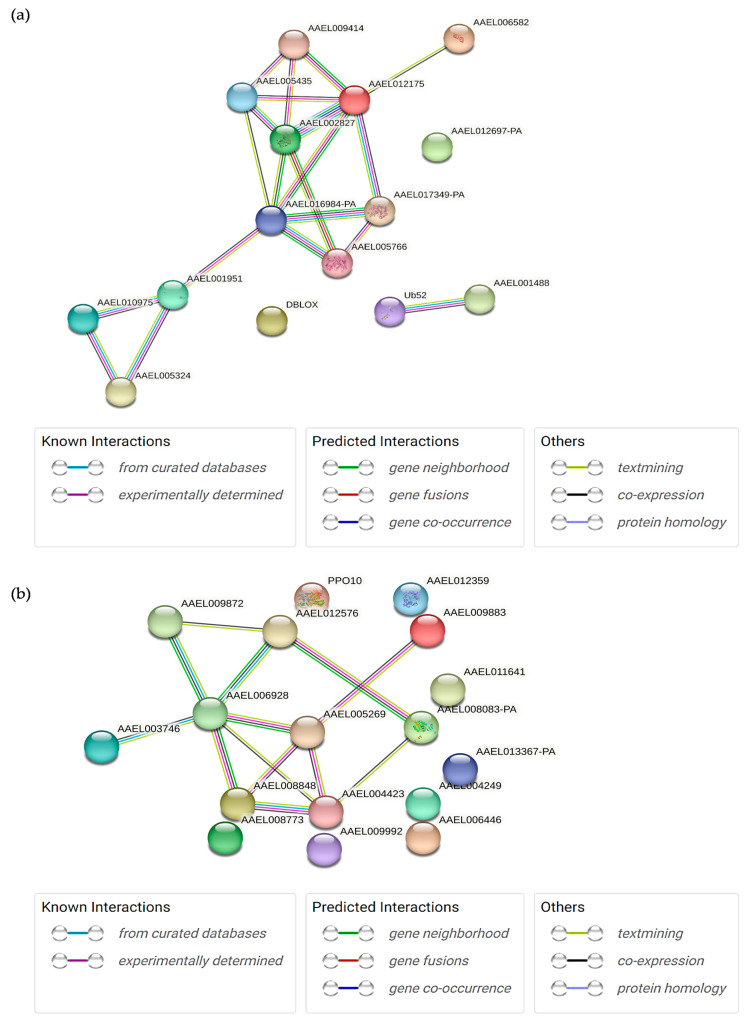
The protein–protein interaction networks of differentially expressed proteins in *Ae. aegypti* (**a**) larvae and (**b**) adults after EA treatment. The colored lines indicate the type of interaction evidence.

To gain a deeper understanding of the molecular mechanism of *Ae. aegypti* in response to the EA extract, the STRING online platform, which determines the interactions between the differentially expressed proteins was used. Two distinct interactions were predicted in the larvae group, where 11 proteins were highly connected to each other (Protein ID: Q17AE9, Q175R3, Q16VZ4, Q1HRQ7, Q17A09, Q17H12, Q1HR69, J9HYM2, Q178U8, Q6QNY2, Q16RF4) (Figure 1a). These proteins collectively function as a whole, with some involved in processes such as calcium ion transport and homeostasis, energy metabolism and ATP synthesis, protein folding and chaperone activity, cell structure, organization, and motility, as well as muscle contraction and structural protein. The significantly expressed proteins in adult mosquitoes formed a large interaction network that consisted of nine proteins (Protein ID: Q16UK8, Q16LP5, Q174D6, Q0IG02, Q16XK3, Q17AK0, Q16UJ3, Q1HR21, A0A6I8TLV1) (Figure 1b). These proteins exhibit functional relationships and closely collaborate to function as a cohesive unit. They are generally involved in various processes, including amino acid metabolism, metabolism and oxidation-reduction reactions, glycolysis and energy metabolism, protein degradation and regulation, mitochondrial ATP synthesis, and ribosome function and protein synthesis.

**Figure 2:**
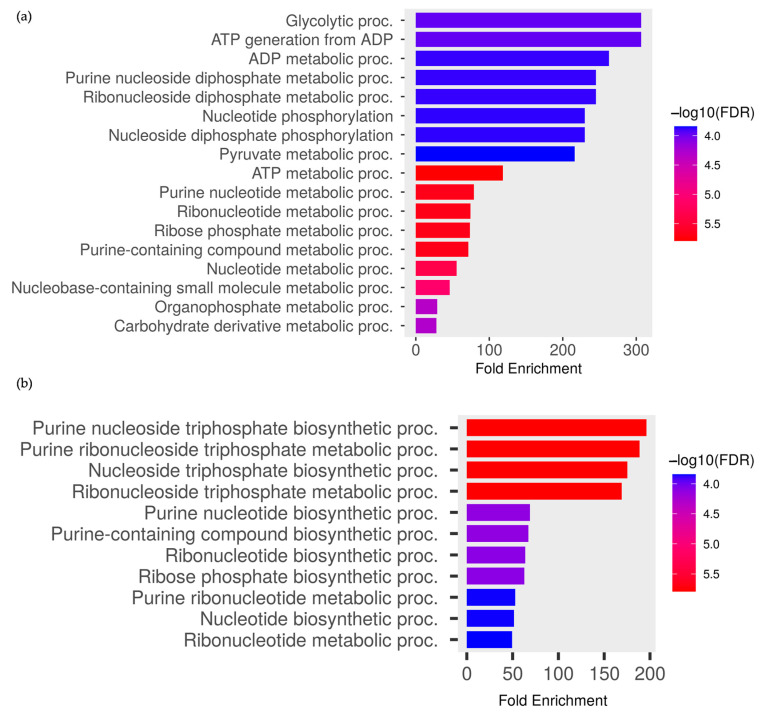
Classification of the significantly expressed proteins by EA extract treatment in relation to the biological process in *Ae. aegypti* (**a**) larvae and (**b**) adults. The categories were derived from the gene ontology analysis of biological processes at the ShinyGo platform.

The gene ontology of differentially expressed larvae proteins in terms of biological processes and cellular components was also analyzed via ShinyGo based on the *Aedes aegypti* LVP_AGWG database. The FDR indicated the likelihood of the enrichment by chance while the fold enrichment showed the overrepresentation of genes of a certain pathway. The GO analysis suggested the proteins that expressed prominently in larvae were involved in 17 biological functions (Figure 2a). The glycolytic process and ATP generation from ADP showed the highest fold enrichment (306.6-fold) between other biological processes, followed by the ADP metabolic process (262.8-fold) and purine nucleoside, as well as the ribonucleoside biphosphate metabolic process (245.3-fold) (Figure 2a). The carbohydrate derivative metabolic processes have the lowest fold enrichment (28-fold) (Figure 2a). The significantly expressed proteins in adults were mostly involved in the purine nucleoside triphosphate biosynthetic process with a fold enrichment of 196.2, followed by the purine ribonucleoside triphosphate metabolic process (188.7-fold) and nucleoside triphosphate biosynthetic process (175.2-fold) (Figure 2b). The ribonucleotide metabolic process had the lowest fold enrichment (49.6-fold) compared to other biological processes (Figure 2b). The larvae and adults exhibited distinct biological profiles, with the larvae primarily focusing on energy production and metabolic processes, specifically related to glycolysis and ATP generation. In contrast, adults exhibit a strong emphasis on nucleotide biosynthesis and related metabolic processes, which may indicate an increased demand for energy production.

**Figure 3:**
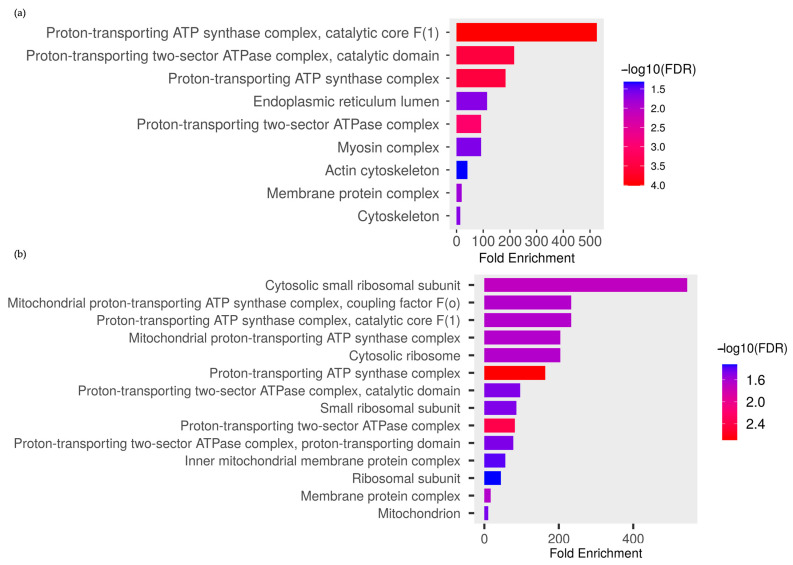
Classification of the significantly expressed proteins by EA extract treatment in relation to the cellular component in *Ae. aegypti* (**a**) larvae and (**b**) adults. The categories were derived from the gene ontology analysis of cellular components at the ShinyGo platform.

Most differentially expressed proteins in the larval stage were located in a proton-transporting ATP synthase complex (Figure 3a), whereas in the adult stage, they were located in a cytosolic small ribosomal subunit, followed by the mitochondrial proton-transporting ATP synthase complex, coupling factor F(0), and catalytic core F(1) (Figure 3b).

To understand the structure–activity relationship between the target mosquito proteins (ATP synthase subunit alpha and beta, ATP citrate synthase, and FBA) and potential bioactive compounds, molecular docking was performed to predict the binding mode and binding affinity of the ligands towards the proteins. The ability of a protein to interact with ligands and form a supramolecular complex held together by noncovalent bonds plays a crucial role in protein dynamics, which may further inhibit or strengthen its biological functions [21]. The AutoDock Vina 1.1.2 software was used to perform the blind molecular docking. Table 2 summarizes the molecular docking results according to the highest binding affinities between each of the bioactive compounds and target proteins. Among all four tested compounds, dodemorph and selagine showed higher binding affinities with the mosquito proteins. The dodemorph and selagine bind strongly towards FBA with binding affinities of −8.0 and −8.1 kcal/mol, respectively, suggesting that FBA might be a potential target for dodemorph and selagine.

**Figure 4:**
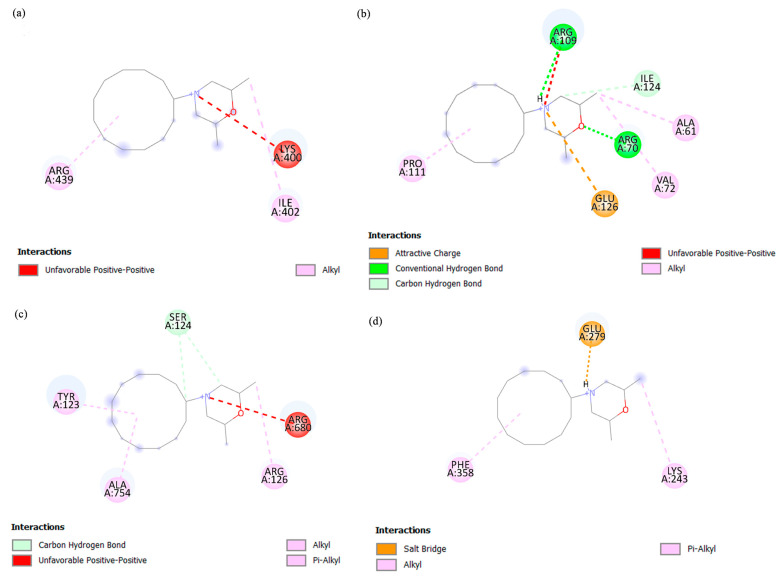
Two-dimensional diagram of the interaction between dodemorph with (**a**) ATP synthase subunit alpha, (**b**) ATP synthase subunit beta, (**c**) ATP citrate synthase, and (**d**) FBA. The diagram visually depicts the ligand–receptor interaction, showcasing distinct colors to represent the various interactions, along with the identification of binding amino acid residues within the protein’s binding pocket.

**Figure 5:**
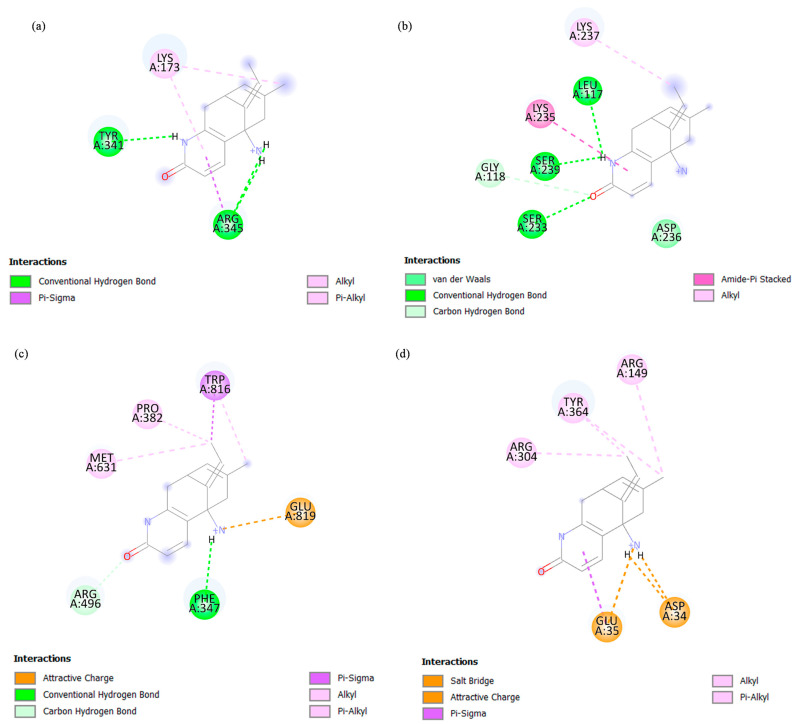
Two-dimensional diagram of the interaction between selagine with (**a**) ATP synthase subunit alpha, (**b**) ATP synthase subunit beta, (**c**) ATP citrate synthase, and (**d**) FBA. The diagram visually depicts the ligand–receptor interaction, showcasing distinct colors to represent the various interactions, along with the identification of binding amino acid residues within the protein’s binding pocket.

Dodemorph and selagine were selected for further visualization and analysis due to their higher binding affinities among other bioactive compounds towards the target mosquito proteins. The 2D binding models between dodemorph and selagine with the mosquito proteins were analyzed using Discovery Studio 3.5 software (Figure 4 and Figure 5). Figure 4 and Figure 5 demonstrate various interactions (alkyl, pi-alkyl, pi-sigma, amide-pi stacked, attractive charge, conventional hydrogen bond, carbon hydrogen bond, salt bridge, van der Waals, and unfavorable positive–positive) involved between the binding residues of mosquito proteins and compounds. Dodemorph exhibits the highest number of bond formations (eight bonds) with the ATP synthase subunit beta, whereas FBA shows the highest number of bond formations with selagine. Notably, dodemorph exhibits unfavorable positive–positive interactions with ATP citrate synthase, ATP synthase subunit alpha, and beta (Figure 4). Conversely, FBA forms bonds with both dodemorph and selagine through a salt bridge involving GLU279, as well as with ASP34 and GLU35, respectively.

## 3. Discussion

The current study revealed that the treatments of both *Ae. aegypti* larvae and adults with EA extract significantly expressed certain proteins, suggesting that these proteins may play a role in activating the mosquito’s defense mechanism against the insecticidal compounds in the extract. Exposure to stress conditions may have significantly increased the expression of heat shock cognate 70 (HSC70) in *Ae. aegypti* larvae. HSC70 is a constitutively expressed molecular chaperon of heat shock protein 70 (HSP70) which plays important roles in cell protection towards various environmental stresses, namely temperature fluctuation, ultraviolet exposure, healthcare treatment, and invasion of pathogens via ensuring proper protein folding, membrane translocation, and protein degradation by targeting them to the lysosome or ubiquitin-proteasome system [22,23,24]. Both HSC70 and HSP70 share a significant sequence homology and tend to co-purify with each other, often suggesting their potential equivalence and functional interchangeability to a large extent, despite exhibiting distinct expression patterns [25,26,27]. Similar results were observed in a recent study where the expression of the HSC70 gene (*Alhsc*70) of the British bug *Apolygus lucorum*, a significant agricultural pest of Bt-cotton [28], was upregulated when exposed to pesticide (cyhalothrin), in both transcriptional and translational levels (*p* < 0.05), indicating that HSC70 was important in the development of insecticide tolerance of *A. lucorum* [29]. Upregulation of HSP70 mRNA expression was also reported when insects were exposed to insecticides [30]. Similarly, the solvent extract of streptomycetes obtained from soil samples was observed to cause significant damage to the cuticles of *Culex pipiens* larvae, which play a crucial role in maintaining water balance, protecting them from desiccation [31,32]. HSP70 is also essential for dehydration tolerance in *Ae. aegypti*; without HSP70, *Ae. aegypti* could only tolerate body water loss of 29% while the controls could survive 36% of their body water loss, which in turn lowered the survival of *Ae. aegypti* [33]. During the period of dehydration, proteins tend to aggregate and possibly undergo denaturation, and phase transitions in the membrane might occur, which in turn affects the cellular ions’ homeostasis and transmembrane proteins conformation [34,35]. Thus, HSC70 may play a crucial role in mitigating cellular stress induced by *Ae. aegypti* approaching its maximum limit of dehydration tolerance.

In larval samples, the protein–protein interaction network analysis revealed that HSC70 was functionally related to GAPDH, ATP synthase subunit alpha, and FBA. GAPDH, besides its role in glycolysis, has been associated with various processes such as cell death regulation, autophagy, DNA repair, and RNA exportation under environmental stress [36]. FBA, on the other hand, plays a vital role in glycolysis and energy production by catalyzing the production of glyceraldehyde 3-phosphate and dihydroxyacetone phosphate. Both FBA and HSC70 are involved in the cellular stress response, which further suggests their potential role in the defense mechanism of *Ae. aegypti* against the insecticidal compounds present in the EA extract [37]. FBA was found to have a five-fold increase in expression in insecticide resistant-cotton aphid, *Aphis gossypii*, suggesting a large amount of energy was consumed in the process of insecticide detoxification [38]. HSC70 might ensure the proper folding of FBA and repair it when damaged as FBA is essential in defense against oxidative stress. Furthermore, the absence of HSC70 resulted in reduced energy metabolism causing premature death in the blood-sucking kissing bug *Rhodnius prolixus*, the vector of Chagas disease [39], suggesting its relationship with proteins involved in ATP synthesis such as ATP synthase [40]. ATP synthase is involved in the catalyzing of ATP production from ADP and phosphate by harnessing the energy from the proton gradients across the mitochondrial inner membrane. ATP synthase was upregulated when *Helicoverpa armigera* was exposed to a pyrethroid insecticide [41]. Similar results were observed in spirotetramat-resistant *A. gossypii* [38]. Likewise, the upregulation of ATP synthase subunit alpha and beta was also observed in the present study. The observed increase in energy metabolism is likely used to compensate for the energy expended in the insecticide detoxification process.

Gene ontology analysis revealed that the EA extract primarily targeted the energy metabolism process in larvae, as evidenced by the highest fold enrichments observed in glycolysis and ATP generation. Increased energy produced was proven necessary in activating defense mechanisms during stress conditions [42]. *Aedes aegypti* larvae showed hypersensitivity towards toxins when the ATP synthase gene was silenced via RNA interference [42]. Besides energy metabolism, the EA extract also interferes with the cell structure and movement of *Ae. aegypti* larvae, for instance, actin. Actin is important in helping insect larvae to overcome intoxication via muscle metabolism, improving muscle stability, and contractility [42]. The cytoskeletal element worked closely with cellular membranes and further promoted a cellular response inducing a defense mechanism [43]. In fact, partial silence of actin also leads to two times higher sensitivity to toxins in *Ae. aegypti* larvae [42]. The upregulation of ribosomal L15, which is involved in protein synthesis, might be engaged in generating a stress-response protein. The gene ontology analysis of cellular components provided further evidence that the majority of significantly expressed proteins primarily functioned within ATP synthase complexes, which play a crucial role in ATP synthesis.

The EA extract treatment in adults led to increased pyruvate kinase activity, promoting glucose conversion to pyruvate and ATP energy generation through glycolysis. This energy was potentially utilized for the production of detoxifying enzymes such as dihydrolipoyl dehydrogenase and 40S ribosomal protein SA. In STRING analysis, pyruvate kinase showed a functional relationship with these enzymes and alanine transaminase, all of which contribute to energy production and metabolic homeostasis in insects. Additionally, the 26S protease (S4) subunit of the 26S proteasome complex, which was significantly upregulated, might play a crucial role in ATP-dependent protein degradation, cell cycle progression, DNA damage repair, transcription, and stress response, ensuring protein homeostasis in eukaryotic cells [44]. The upregulation of 26S protease suggested an increase in degradation products (amino acids) that might be utilized in the synthesis of proteins incorporated in defensive systems during stress in mosquitoes [45]. However, a previous study reported the downregulated 26S proteasome non-ATPase regulatory subunit in *Anopheles stepehensi* multi-insecticide resistant strain, with a fold expression value of 0.25 compared to the susceptible strain, which is contradicted by the result obtained in this study [46]. Consequently, when exposed to various pesticides across different insect species, distinct regulatory mechanisms may be observed in pesticide-responsive proteins.

The 40S ribosomal proteins S3a and SA were significantly expressed after the EA extract treatment in adult *Ae. aegypti*. Both proteins are the component of a small ribosomal subunit which is involved in triggering protein synthesis after binding on the initiation factor of messenger RNA. However, their expressions exhibited contrasting patterns. The subunit S3a was downregulated while the subunit SA was upregulated based on the heatmap analysis. Previous studies reported an elevation in the expression of ribosomal proteins in insecticidal-resistant strains [47,48,49]. The enhancement of ribosome production could increase the translational capacity and stress-response protein generation that may be involved in insecticide detoxification [50]. Nevertheless, the alanine transaminase (ALT) notably reduced expression level with the lowest fold-change compared to other proteins after the EA treatment in adult mosquitoes. Similar results were observed in *A. gossypii* adults where the ALT activity was significantly lowered by methanol extract from entomopathogenic fungi [51]. Lower activities of detoxification enzymes including ALT were also observed in honey bees *Apis mellifera*, which might result in the accumulation of harmful agents such as peroxides and free radicals, leading to cellular oxidative damage [52]. Most significantly expressed proteins were involved in the purine nucleoside and ribonucleoside triphosphate biosynthesis process, which are responsible for many cellular processes, including energy metabolism, signaling, and DNA and RNA synthesis [53]. The outcomes from the gene ontology analysis provided additional confirmation that the EA extract markedly impacted the proteins that contributed to energy metabolism.

Both larval and adult groups shared three significantly expressed proteins, namely ATP synthase, FBA, and ATP citrate synthase, which were upregulated after treatment. Studies have reported that the natural product of streptomycetes exhibits physiological effects on mitochondria. For instance, the bacteria *Streptomyces hygroscopicus* var. *ossamyceticus* produces ossamycin, which targets the mitochondrial F1F0-type ATP synthase to prevent electron transport [54]. The beta subunits of ATP synthase that are directly responsible for ATP production also showed an increase in expression in *Ae. aegypti* larvae when exposed to LC_10_ of toxin dose [42]. This finding aligned with the protein expression observed in both the larvae and adults of *Ae. aegypti*. The ATP synthase is essential to activate defense mechanisms against stress conditions via energy production in *Ae. aegypti* [42]. Likewise, larvae with defective ATP synthase also exhibited hypersensitivity characteristics towards toxins [42]. Oxidative stress which might be induced by insecticide could promote the upregulation of FBA in both larval and adult stages of insects [55]. The ATP citrate synthase is involved in the catalytic process of the conversion of acetyl-CoA and oxaloacetate into acetate in the Krebs cycle, which produces aerobic energy and promotes metabolites conversion [56]. An increase in the activity of citrate synthase could indicate an increase in mitochondrial activity that could potentially aid in the detoxification of insecticides, as detoxification pathways often require energy. The observed differences between larvae and adults in their biological profiles based on gene ontology analysis might be primarily attributed to their developmental changes and specific physiological requirements. These factors play a crucial role in shaping their distinct responses and adaptations to the EA extract, allowing them to survive and fulfill their respective life stage functions.

The docking results showed that the bioactive compounds may potentially influence protein expression due to their observed interactions with the protein. Based on the highest binding affinities, dodemorph and selagine are more likely to interact strongly with the mosquito proteins as they have high binding affinities. The strength of the compound binding to the protein’s binding site is directly proportional to higher binding affinities [57]. The observation of unfavorable positive–positive interactions suggests that ATP citrate synthase, ATP synthase subunit alpha, and beta may not be suitable protein targets for dodemorph. Indeed, unfavorable bonds can compromise the stability of drugs, potentially leading to repulsive forces between the ligand and proteins [58]. The presence of a salt bridge is considered crucial in ligand interactions, as inhibiting the formation of a salt bridge between the ligand and protein has been observed to significantly diminish the potency of enzyme inhibitors [59]. Among the proteins examined, only FBA exhibited a salt bridge interaction with dodemorph and selagine, implying that FBA may be a potential target for the EA extract. Compared to salt bridges formed on the surface of the proteins, buried salt bridges can remarkably contribute to ligand binding [60]. In this aspect, it is noteworthy that selagine forms salt bridges with FBA amino acid residues ASP34 and GLU35, while dodemorph interacts with GLU279 within the protein’s catalytic center. These salt bridge interactions, occurring internally, likely possess significant strength, which could potentially contribute to the high binding affinities observed between the FBA and dodemorph or selagine.

## 4. Materials and Methods

### 4.1. Colonization of Aedes aegypti Larvae and Adults

*Aedes aegypti* adults were kept in a 30 cm × 30 cm × 30 cm cage covered with netting and fed with 10% sucrose solution as a food source [61]. The four- to five-day-old female mosquitoes were provided with a blood meal using a Hemotek membrane feeding system to produce the F1 generation that was used for subsequent bioassay. A plastic cup containing 200 mL chlorine-free water and filter paper was placed in the cage for egg collection. The collected eggs were submerged in a plastic container filled with chlorine-free water for hatching into larvae. To accommodate *Ae. aegypti* feeding patterns, beef liver powder, as a food source, was placed at the bottom corner of the container. Subsequently, after larvae had grown into pupae, the pupae were moved into a small plastic cup filled with dechlorinated water and further introduced into the rearing cage for adult emergence.

### 4.2. Fermentation and Extraction of Bioactive Compounds from Streptomyces *sp.* KSF103

*Streptomyces* sp. KSF103 was isolated from soil samples in Pahang, Malaysia and it was further maintained on International *Streptomyces* Project 2 agar (ISP2) at 28 °C. To prepare the bioactive secondary metabolites, the *Streptomyces* sp. KSF103 sample was inoculated in the ISP2 broth with pH 7.2 under the aseptic technique and incubated at 28 °C for 14 days on a shaker incubator at a speed of 110 RPM [20]. After 14 days, the medium was collected and centrifuged at 5000 RPM for 5 min to obtain the culture supernatant. The collected supernatant was then added with an equal volume of EA (Fulltime, Anqing, China). The mixture was shaken intermittently and continuously for three days to separate the secondary metabolites from the culture broth. To extract the secondary metabolites, the EA solvent was concentrated using a rotary evaporator. The collected EA extract was stored at −20 °C for future tests.

### 4.3. Larval and Adult Bioassays

A total of 25 late third or early fourth instar larvae were transferred into a cup containing 100 mL of water [62]. To avoid undue mortality, the water level in the cups remained between 5 and 10 cm [62]. The larvae were exposed to a lethal concentration of 0.04 mg/mL, which corresponds to the LC_50_ for larvae based on [20]. A 0.01 g of EA extract was first added with 0.5 mL of dimethyl sulfoxide (DMSO) (Sigma-Aldrich, Burlington, MA, USA) to achieve a concentration of 20 mg/mL before further diluting it to LC_50_ with distilled water. DMSO was used as a negative control. The treated larvae were held at 28 °C on a 12 h day/12 h night cycle. The larval mortality was recorded 24 h post-exposure.

The insecticidal activity of *Streptomyces* sp. KSF103 EA extract on adults was determined via a direct topical application assay [63]. The three- to five-day-old female mosquitoes were collected for adult bioassay using an aspirator. The treatment group received a dose of 30.1 mg/mL LC_50_, which was determined based on prior research conducted by [20]. Acetone was used as a negative control (R&M Chemicals, Subang Jaya, Malaysia). Briefly, the test solution was applied to the dorsal thorax of anaesthetized mosquitoes using a syringe micro applicator. The treated mosquitoes were then kept under constant conditions of 28 °C and 7585% humidity on a 12 h day/12 h night cycle. The mosquitoes’ mortality was recorded 24 h post-exposure. The mosquitoes were considered dead if they could not stand.

A total of 20 larvae/adults from each group were further subjected to shotgun protein identification analysis.

### 4.4. Shotgun Proteomics

The protein profile of treated and untreated (control) *Ae. aegypti* in both larval and adult stages were analyzed via liquid chromatography with tandem mass spectrometry (LC-MS/MS). LC-MS/MS analysis is a superior approach compared to other methods due to its high analytical sensitivity and specificity along with reduced sample preparation requirements [64]. Additionally, LC-MS/MS exhibits a wide dynamic range, allowing for accurate quantification across a broad concentration spectrum [64,65]. The samples were first dipped into a small canister containing liquid nitrogen for a few seconds. They were then ground to powder using sterile tips. The samples were kept at −80 °C until further analysis. To prepare the protein samples for LC-MS/MS analysis, an appropriate amount of lysis buffer containing protease inhibitors was first added to the protein samples, and sonication was performed to aid in cell lysis and protein extraction. After centrifugation, the supernatant, containing the protein extract, was transferred to a new tube. The protein concentration was determined using a BCA kit, and an equal amount of protein was precipitated with methanol and chloroform. The precipitated proteins were dissolved in a urea solution and subjected to denaturation and alkylation. Ammonium bicarbonate was added to adjust the pH, and trypsin was used for digestion. The resulting peptides were purified with a C18 SPE column to remove salt, followed by lyophilization. Prior to LC-MS/MS analysis, the peptides were resuspended in 0.1% formic acid. These steps ensured the proper preparation of the protein samples for subsequent LC-MS/MS analysis.

The nanoLC setup used the Ultimate 3000 nano UHPLC system from ThermoFisher Scientific (Waltham, MA, USA). It consisted of a trapping column (PepMap C18) and an analytical column (PepMap C18) for sample separation. A 1 μg sample was loaded onto the system, and a mobile phase comprising water with 0.1% formic acid (A) and 80% acetonitrile with 0.1% formic acid (B) was used. The LC linear gradient involved gradually increasing the concentration of buffer B over time. The mass spectrometry analysis included a full scan between 300 and 1650 *m*/*z* at a resolution of 60,000 at 200 *m*/*z*. The MS/MS scan operated in Top 20 mode, selecting the most intense ions for fragmentation. Various parameters were set, such as collision energy, isolation window, charge state exclusion, and dynamic exclusion to optimize data acquisition.

### 4.5. Data Analyses

Four raw MS files obtained from the proteomics analysis were analyzed and searched against the *Ae. aegypti* protein database using Maxquant (1.6.2.14) [66]. Some parameters were considered as follows during the algorithm analysis: the protein modification involved setting carbamidomethylation as a constant modification while oxidation was considered a variable modification; trypsin was selected for the enzyme specificity; the maximum missed cleavages were set as 2; the precursor ion mass tolerance was adjusted to 10 ppm; and the MS/MS tolerance was 0.6 Da [67]. Venn diagrams were produced from the raw data obtained from LC-MS/MS analysis using the Maxquant algorithm. The raw data were then filtered based on the protein ID score. Proteins with scores of more than 10 were chosen for heatmap analysis [68]. A heatmap of log_2_fold change between the treated and untreated groups was plotted. The fold change and log_2_fold change were calculated based on the change in protein intensity in treated mosquitoes. Proteins with a log_2_fold change greater than 1.5 were considered significantly upregulated while those proteins with a log_2_fold change less than −1.5 were known to be significantly downregulated [69]. The gene symbol, protein name, and molecular functions of the significantly expressed proteins were discovered from UniProtKB. STRING (Search Tool for the Retrieval of Interacting Genes/Proteins) was utilized to identify the significantly expressed protein–protein interactions. Gene ontology analysis of significantly regulated proteins in terms of biological processes and cellular components was carried out using ShinyGo [70]. ShinyGo utilizes the false discovery rate (FDR) to assess the likelihood of enrichment occurring due to chance.

### 4.6. Identification of Bioactive Compounds of EA Extract

The potential bioactive compounds were further identified via liquid chromatography–mass spectrometry (LCMS). The compounds were matched via the Metlin database. The identified compounds from LCMS were proposed compounds for a specific mass-to-charge (*m*/*z*) value as there might be multiple compounds sharing the same *m*/*z* value. To narrow down the data given in the compound list folder, only compounds with Score Db or Score MFG close to 100, Diff(Db.ppm) or Diff(MFG.ppm) values within −2 to +2, and did not appear in blanks were selected [71]. Db represented the compound matched against the Metlin database. At the same time, MFG was the algorithm used by the software to calculate the values if the compound did not appear in the database. The filtered LCMS data determined four bioactive compounds, namely C17 sphinganine, dodemorph, metyrapol, and selagine.

### 4.7. In Silico Molecular Docking

The potential insecticidal activity of EA extract was confirmed via molecular docking to analyze the binding affinity, confirmation, and pattern with the *Ae. aegypti* proteins. The common proteins between larval and adult samples (ATP synthase (alpha and beta subunit), FBA, and ATP citrate synthase) were further selected as potential targets for the bioactive compounds identified from the EA extract. The three-dimensional (3D) structures of the common proteins were modeled using the Iterative Threading Assembly Refinement (I-TASSER) platform according to the amino acid sequences retrieved from Universal Protein Resource (UniProt) [72]. Using Swiss PDB Viewer 4.1.0 software [73], the protein structures were minimized using GROMOS 43B1 forcefield. The crystallographic waters and all non-standard residues were removed from the potential target protein structures using the UCSF Chimera [74]. The 3D compound structure was downloaded from the PubChem server [75]. The compound structure was energy minimized using UCSF Chimera with default AMBER force field (AMBERff14Sb). Prior to docking, the preparation of both the compounds and proteins by adding hydrogen molecules using UCSF Chimera was saved as PDBQT files. Blind docking was performed in which the entire protein of interest was covered in a grid box using AutoDock 1.5.6 software [76]. The spacing size of grid boxes generated for all the proteins was set at 1.000 Å and the parameters used for molecular docking of four of the bioactive compounds with each protein of interest using AutoDock Vina 1.1.2 software [77] are tabulated in Table 3. Following the docking process, the output PDBQT files were split into a single file via the Vina_split option in AutoDock Vina 1.1.2 software for further analysis. The binding affinities, type of bonding, and close contact residues from each conformation were analyzed using AutoDock Vina 1.1.2 and Discovery Studio 3.5 software.

## 5. Conclusions

Proteomics analysis of larvae and adult *Ae. aegypti* highlighted how *Streptomyces* sp. KSF103 EA extract interacts with the mosquito proteins for its insecticidal properties. The results showed that the extract mainly tackled the proteins involved in energy metabolism in larval and adult stages of *Ae. aegypti*, suggesting it might be related to the insecticide detoxification process. The larval and adult mosquitoes shared three significantly expressed proteins that are mainly involved in defense mechanism activation during treatment. In addition, this study further supported the potential of EA extract in limiting the survivability of *Ae. aegypti* reported in a previous study. The interaction between the compound and the proteins provides preliminary evidence suggesting that the extract compounds, specifically dodemorph and selagine, may potentially affect the protein expression in mosquitoes. Furthermore, FBA might serve as a prospective target protein for the insecticidal properties of the EA extract. However, it is crucial to conduct additional experimental validation to confirm the effect of the compound on protein expression via Western blot. Future studies will be directed toward the identification of specific compounds that may limit the survivability of *Ae. aegypti* via *in vitro* and *in vivo* assays.

## Figures and Tables

**Table 1:** Significantly expressed proteins with their respective gene symbol, protein name, and molecular function in *Ae. aegypti* larvae and adults.

Protein IDs	Gene Symbol	Protein Name	Molecular Function
Larvae			
Q1HR69	23687769	Heat shock cognate 70	ATP binding and ATP-dependent protein folding chaperone
Q7KF35	Ub52	Ubiquitin-60S ribosomal protein L40	Structural constituent of ribosome
Q6QNY2	5572985	Actin	ATP binding, hydrolase activity, identical protein binding, and structural constituent of cytoskeleton
Q17H12	5576214	ATP synthase subunit beta	ATP binding, proton-transporting ATP synthase activity, rotational mechanism, and proton-transporting ATPase activity, rotational mechanism
Q178U8	5567031	Fructose-bisphosphate aldolase	Fructose-biphosphate aldolase activity
Q175R3	AAEL006582	Calcium-transporting ATPase	ATP binding, ATP hydrolysis, and P-type calcium transporter activity
Q17L12	AAEL001488	Ribosomal protein L15	Structural constituent of ribosome
J9HYM2	23687404	Glyceraldehyde-3-phosphate dehydrogenase	Glyceraldehyde-3-phosphate dehydrogenase (NAD+) (phosphorylating) activity, NAD binding and NADP binding
Q17AE9	AAEL005324	AAEL005324-PA	ATP binding
Q16RF4	5574161	Paramyosin, long form	-
Q16VZ4	5571907	NADH-ubiquinone oxidoreductase 39 kda subunit	Protein-containing complex binding
Q17A09	AAEL005435	AAEL005435-PB	Metal ion binding and metalloendopeptidase activity
Q1DGF0	AAEL012697	AAEL012697-PA	-
Q0IFX2	DBLOX	AAEL003933-PA	Heme binding and peroxidase activity
Q1HRQ7	5575914	ATP synthase subunit alpha	ATP binding and proton-transporting ATP synthase activity, rotational mechanism
Adults			
Q16LP5	AAEL012576	Pyruvate kinase	ATP binding, kinase activity, magnesium ion binding, potassium ion binding, and pyruvate kinase activity
Q16U81	AAEL009992	AAEL009992-PA	-
Q16UJ3	5572552	26S protease (S4) regulatory subunit, putative	ATP binding and ATP hydrolysis activity
Q16P45	PPO10	AAEL011764-PA	Monooxygenase activity
Q17DD5	5564443	Uncharacterized protein	-
Q16PI3	5575085	Transferrin-like domain-containing protein	-
Q174D6	AAEL006928	Dihydrolipoyl dehydrogenase	Dihydrolipoyl dehydrogenase activity and flavin adenine dinucleotide binding
Q17AK0	AAEL005269	AAEL005269-PA	Metal ion binding
Q176D3	5568007	Uncharacterized protein	Trehalose-phosphatase activity
Q16JD9	AAEL013367	AAEL013367-PA	Chitin binding and hydrolase activity, acting on carbon-nitrogen (but not peptide) bonds
Q16MB4	5576170	Nucleoside diphosphate kinase	ATP binding and nucleoside diphosphate kinase activity
Q1HR21	5564798	ATP synthase subunit d, mitochondrial	Proton transmembrane transporter activity
A0A6I8TLV1	5570108	40S ribosomal protein SA	Structural constituent of ribosome
Q16XK3	5571150	ATP synthase subunit gamma	Proton-transporting ATP synthase complex, catalytic core F(1)
Q0IG02	5578926	Acetyl-CoA hydrolase	Acetate CoA-transferase activity and acetyl-CoA hydrolase activity
Q16XT3	AAEL008773	AAEL008773-PA	-
Q0IFA5	RS3A_AEDAE_a	40S ribosomal protein S3a	Structural constituent of ribosome
Q16UK8	AAEL009872	Alanine transaminase	Pyridoxal phosphate binding and transaminase activity

**Table 2:** The highest binding affinities of bioactive compounds (C17 sphinganine, dodemorph, metyrapol, and selagine) when docked against different mosquito proteins using AutoDock Vina 1.1.2.

Proteins	Binding Affinity (kcal/mol)			
	C17 Sphinganine	Dodemorph	Metyrapol	Selagine
ATP synthase subunit alpha	−3.9	−6.1	−5.4	−6.3
ATP synthase subunit beta	−4.0	−6.8	−5.9	−7.1
ATP citrate synthase	−4.1	−6.7	−5.6	−6.7
FBA	−4.9	−8.0	−6.7	−8.1

**Table 3:** Parameters used for molecular docking of the bioactive compounds (C17 sphinganine, dodemorph, metyrapol, and selagine) with selected mosquito proteins.

Proteins	Center-X	Center-Y	Center-Z	Size-X	Size-Y	Size-Z
ATP synthase subunit alpha	69.954	84.147	85.130	82	104	84
ATP synthase subunit beta	80.289	77.239	79.967	60	50	102
ATP citrate synthase	99.896	94.484	111.176	80	98	126
FBA	66.379	65.163	68.427	56	54	58

## Data Availability

Not applicable.

## Supplementary Materials

The following supporting information can be downloaded at: https://www.mdpi.com/article/10.3390/ijms241512398/s1.
