# Supplementary material for: Protein Profiling of Aedes aegypti Treated with Streptomyces sp. KSF103 Ethyl Acetate Extract Reveals Potential Insecticidal Targets and Metabolic Pathways
Source: Int J Mol Sci. 2023 Aug 3;24(15):12398. doi: 10.3390/ijms241512398 (PMC10418484; doi:10.3390/ijms241512398)

Supplementary Table S1: Differential regulation of proteins identified by shotgun proteomic analysis with fold change in *Ae. aegypti* larvae and adults after the treatment of *Streptomyces* sp. KSF103. EA extract. The asterisk indicated the significantly expressed proteins with log $_2$ fold change ≤ -1.5 or ≥ 1.5.

Larvae

Protein IDs	Protein Name	Fold Change	Log$_2$ Fold Change
Q1HR69	Heat shock cognate 70	16.46	4.04*
Q7KF35	Ubiquitin-60S ribosomal protein L40	12.41	3.63*
Q6QNY2	Actin	10.97	3.46*
Q17H12	ATP synthase subunit beta	9.00	3.17*
Q178U8	Fructose-bisphosphate aldolase	7.04	2.82*
Q175R3	Calcium-transporting ATPase	5.65	2.50*
Q17L12	Ribosomal protein L15	5.61	2.49*
J9HYM2	Glyceraldehyde-3-phosphate dehydrogenase	5.18	2.37*
Q17AE9	AAEL005324-PA	4.90	2.29*
Q16RF4	Paramyosin, long form	4.57	2.19*
Q16VZ4	NADH-ubiquinone oxidoreductase 39 kda subunit	3.79	1.92*
Q17A09	AAEL005435-PB	3.40	1.77*
Q1DGF0	AAEL012697-PA	3.21	1.68*
Q0IFX2	AAEL003933-PA	2.88	1.52*
Q1HRQ7	ATP synthase subunit alpha	2.86	1.52*
Q17GM7	Probable citrate synthase 1, mitochondrial	2.38	1.25
A0A6I8TS47	Basement membrane-specific heparan sulfate proteoglycan core protein	2.36	1.24
Q16PI3	Transferrin	1.58	0.66
Q17EE8	Histone H4	1.54	0.62
Q16V54	Serine collagenase 1 precursor, putative	1.33	0.41
Q1HR88	Elongation factor 1-alpha	0.83	-0.27
Q176H2	AAEL006368-PA	0.70	-0.52

Adults

Q16LP5	Pyruvate kinase	12.34	3.62*
Q16U81	AAEL009992-PA	8.27	3.05*
Q16UJ3	26S protease (S4) regulatory subunit, putative	5.72	2.52*
Q16P45	AAEL011764-PA	5.46	2.45*
Q17DD5	Uncharacterized protein	4.65	2.22*
Q16PI3	Transferrin-like domain-containing protein	4.63	2.21*
Q174D6	Dihydrolipoyl dehydrogenase	3.96	1.99*
Q17AK0	AAEL005269-PA	3.94	1.98*
Q176D3	Uncharacterized protein	3.92	1.97*
Q16JD9	AAEL013367-PA	3.78	1.92*
Q16MB4	Nucleoside diphosphate kinase	3.69	1.89*
Q1HR21	ATP synthase subunit d, mitochondrial	3.14	1.65*
A0A6I8TLV1	40S ribosomal protein SA	3.10	1.63*
Q16XK3	ATP synthase subunit gamma	3.00	1.59*
Q0IG02	Acetyl-CoA hydrolase	2.94	1.55*
Q16XT3	AAEL008773-PA	2.82	1.50*
Q1HRR9	Peptidyl-prolyl cis-trans isomerase	2.75	1.46
Q6QNY2	Pupal-specific flight muscle actin	2.68	1.42
Q17H12	ATP synthase subunit beta	2.67	1.42
Q1HQJ0	60S ribosomal protein L4	2.62	1.39
Q16EL0	AAEL015116-PA	2.55	1.35
Q17BX4	Uncharacterized protein	2.53	1.34
Q1HRH7	Catalase	2.50	1.32
Q16P06	AAEL011802-PA	2.49	1.31
Q1HR13	Prohibitin	2.42	1.27
Q16HQ0	AAEL000419-PA	2.36	1.24

Q17P76	Fructose-bisphosphate aldolase	2.30	1.20
J9HT37	Phosphotransferase	2.25	1.17
Q0IFX2	AAEL003933-PA	2.19	1.13
Q176Z3	AAEL006222-PA	2.13	1.09
A0A6I8U5Z1	Uncharacterized protein	2.12	1.09
Q1HRT9	40S ribosomal protein S5	2.09	1.07
A0A6I8T8L2	Ubiquitin-like domain-containing protein	2.09	1.06
Q16RV8	AAEL010833-PA	2.08	1.06
Q17NB9	AAEL000749-PA	2.04	1.03
A0A6I8T7P8	Cytochrome c oxidase subunit 4	2.00	1.00
Q1HR77	Ribosomal protein L3	1.99	0.99
Q17D16	AAEL004369-PA	1.98	0.98
Q17HE2	AAEL002720-PA	1.95	0.97
Q1HR32	60S ribosomal protein L8	1.92	0.94
Q177P3	Phosphoglycerate mutase	1.91	0.93
Q5MM88	Cytochrome b-c1 complex subunit 7	1.87	0.90
Q16SE5	1,4-alpha-glucan branching enzyme	1.85	0.89
Q1HRL6	NADH-ubiquinone oxidoreductase 24 kda subunit	1.83	0.87
A0A6I8U4L8	PDZ domain-containing protein	1.81	0.86
A0A6I8TLR5	NADH dehydrogenase [ubiquinone] iron-sulfur protein 3, mitochondrial	1.79	0.84
A0A6I8TII1	Glycogen debranching enzyme	1.77	0.82
Q16PR4	AAEL011551-PA	1.75	0.80
Q17A27	Multifunctional fusion protein	1.73	0.79

Q1HQU4	Saccharopine dehydrogenase domain-containing protein	1.72	0.78
Q17A03	Alpha-galactosidase	1.72	0.78
A0A6I8TGV5	Uncharacterized protein	1.71	0.78
Q16IU1	AAEL013559-PA	1.71	0.78
Q1HRQ7	ATP synthase subunit alpha	1.71	0.77
A0A0P6IYG4	ADP/ATP translocase	1.70	0.77
Q17G59	AAEL003194-PA	1.70	0.76
A0A6I8T9C7	ATP-citrate synthase	1.66	0.73
Q17CT0	Transketolase-like pyrimidine-binding domain-containing protein	1.65	0.72
O16109	V-type proton ATPase catalytic subunit A	1.62	0.69
Q17HW3	Triosephosphate isomerase	1.60	0.68
Q17B69	AAEL005054-PA	1.59	0.66
Q1HQI0	39S mitochondrial ribosomal protein L28	1.58	0.66
Q16YD0	Acyl-coenzyme A oxidase	1.57	0.65
A0A1S4FHF8	Malate dehydrogenase	1.57	0.65
Q9BME7	Elongation factor 2	1.55	0.64
Q175A4	oxoglutarate dehydrogenase (succinyl-transferring)	1.54	0.62
Q17A09	AAEL005435-PA	1.54	0.62
Q16LR5	NADH-ubiquinone oxidoreductase 75 kDa subunit, mitochondrial	1.53	0.62
Q1HRQ9	40S ribosomal protein S8	1.52	0.60
Q16YC2	AAEL008607-PA	1.49	0.57
A0A1S4EXR8	Malic enzyme	1.46	0.55
Q179J9	ATP synthase subunit b	1.45	0.54

Q9XYC8	Vacuolar proton pump subunit B	1.45	0.53
Q16GA3	AAEL014449-PA	1.42	0.51
A0A6I8T9G2	Dihydrolipoamide acetyltransferase component of pyruvate dehydrogenase complex	1.41	0.50
Q16TI5	NADH dehydrogenase [ubiquinone] 1 beta subcomplex subunit 9	1.41	0.50
Q1HR08	Mitochondrial cytochrome c1	1.41	0.49
Q1HR38	ATP synthase subunit	1.41	0.49
Q16Q68	Complex I-49kD	1.39	0.47
A0A1S4FIY1	Malate dehydrogenase, mitochondrial	1.39	0.47
A0A6I8T6L7	Twitchin	1.39	0.47
Q17DN4	AAEL004112-PA	1.37	0.45
Q17KS3	Glycerol-3-phosphate dehydrogenase [NAD(+)]	1.36	0.45
Q1HR81	60S ribosomal protein L7	1.36	0.44
Q17G27	AAEL003235-PA	1.35	0.44
Q1HRD1	C-Type Lectin	1.35	0.43
Q16TK2	Myofilin	1.31	0.39
Q16IX7	AAEL013515-PA	1.30	0.37
Q17IM5	Uncharacterized protein	1.29	0.36
Q16XK7	Uncharacterized protein	1.26	0.34
A0A6I8TFZ7	fumarate hydratase	1.26	0.33
A0A1S4FPC9	Tox-SGS domain-containing protein	1.26	0.33
Q17M16	AAEL001194-PA	1.25	0.32
Q17NG8	Alpha-1,4 glucan phosphorylase	1.24	0.31
A0A6I8TNR3	Proline dehydrogenase	1.23	0.30

Q16KR4	Aconitate hydratase, mitochondrial	1.22	0.29
Q172T4	AAEL007306-PA	1.22	0.28
Q17GL0	AAEL002978-PA	1.21	0.28
J9HXZ8	AAEL001061-PC	1.21	0.28
Q17H80	AAEL002759-PA	1.20	0.26
Q0IED0	Isocitrate dehydrogenase-like domain-containing protein	1.19	0.25
A0A6R5IQX6	Uncharacterized protein	1.18	0.24
Q17H31	AAEL002776-PA	1.17	0.23
Q17N85	Ubiquitin-activating enzyme E1 C-terminal domain-containing protein	1.16	0.22
A0A6I8TJS2	Succinate dehydrogenase [ubiquinone] flavoprotein subunit, mitochondrial	1.16	0.21
Q1HQN2	Malic enzyme	1.14	0.18
Q16V52	Pyruvate carboxylase	1.12	0.17
Q1HRJ0	Cytochrome c oxidase subunit 5A, mitochondrial	1.12	0.16
Q16K65	Proteasome subunit alpha type	1.12	0.16
A0A6I8U7E0	acetyl-CoA carboxylase	1.10	0.14
J9HYM2	Glyceraldehyde-3-phosphate dehydrogenase	1.10	0.13
Q16LN3	Aspartate aminotransferase	1.09	0.12
Q17AE9	AAEL005324-PA	1.08	0.11
Q1HR24	40S ribosomal protein S14	1.08	0.11
Q1HRE7	Bacteria-responsive protein 1 AgBR1	1.06	0.08
A0A6I8T8S4	Uncharacterized protein	1.06	0.08
Q16UT3	AAEL009795-PA	1.03	0.04
Q1HQQ9	Hypothetical conserved protein	1.03	0.04

Q17GM7	Probable citrate synthase 1, mitochondrial	1.02	0.02
Q16EQ1	Uncharacterized protein	1.01	0.02
Q16Y94	Uncharacterized protein	1.01	0.02
A0A6I8TQI9	EGF-like domain-containing protein	1.01	0.01
Q0IEU5	AAEL008006-PA	1.00	0.01
Q16G96	AAEL014456-PA	0.99	-0.01
Q178J6	Spectrin beta chain	0.99	-0.02
Q16QR2	Peptidase M20 dimerisation domain-containing protein	0.98	-0.03
Q1HR83	peptidylprolyl isomerase	0.96	-0.06
Q17HP8	Peptidase S1 domain-containing protein	0.95	-0.07
Q1HQT6	Vacuolar ATP synthase subunit E	0.94	-0.09
Q17EW2	AAEL003658-PA	0.92	-0.12
Q17B17	AAEL005129-PA	0.90	-0.15
Q17PZ2	Complex III subunit 9	0.89	-0.16
Q178U9	Fructose-bisphosphate aldolase	0.87	-0.20
Q17P80	Isocitrate dehydrogenase [NAD] subunit, mitochondrial	0.82	-0.28
Q16J86	Catalase	0.81	-0.30
A0A6I8U458	Myosin heavy chain, muscle	0.78	-0.35
Q16Z50	Calponin	0.77	-0.37
A0A0N8ERX8	Putative ubiquinone oxidoreductase ndufs8/23 kDa subunit	0.74	-0.44
Q1HR10	Phosphoglycerate dehydrogenase	0.71	-0.49
J9EA44	AAEL007633-PB	0.70	-0.51
Q0IGD8	Uncharacterized protein	0.69	-0.53
A0A6I8TFM2	Integrin alpha-2 domain-containing protein	0.68	-0.55

Q16YQ8	Cystathionine beta-synthase	0.67	-0.57
Q16PB5	Heat shock protein 83	0.65	-0.61
Q173T1	Pyrroline-5-carboxylate reductase	0.65	-0.62
Q16W59	AAEL009324-PA	0.65	-0.62
Q17E81	Glycerol-3-phosphate dehydrogenase	0.64	-0.65
Q0IGD5	Uncharacterized protein	0.36	-1.46
Q0IFA5	40S ribosomal protein S3a	0.32	-1.63*
Q16UK8	Alanine transaminase	0.29	-1.79*

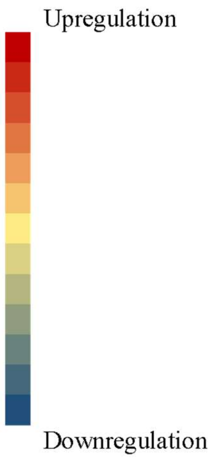

Supplement: Supplementary file 1 [file ijms-24-12398-s001.zip › ijms-2446507-supplementary.pdf]
